# Antimicrobial Active Packaging including Chitosan Films with *Thymus vulgaris* L. Essential Oil for Ready-to-Eat Meat

**DOI:** 10.3390/foods5030057

**Published:** 2016-08-29

**Authors:** Jesús Quesada, Esther Sendra, Casilda Navarro, Estrella Sayas-Barberá

**Affiliations:** IPOA Research Group, Agro-Food Technology Department, School of Engineering of Orihuela, Miguel Hernandez University, Ctra. De Beniel km 3.2. 03312 Orihuela, Alicante, Spain; jesusquesadar@gmail.com (J.Q.); esther.sendra@umh.es (E.S.); casilda.navarro@umh.es (C.N.)

**Keywords:** active packaging, essential oils, thyme, chitosan, ready-to-eat meat, antimicrobial

## Abstract

An active packaging system has been designed for the shelf life extension of ready to eat meat products. The package included an inner surface coated with a chitosan film with thyme essential oil (0%, 0.5%, 1%, and 2%) not in direct contact with the meat. Our aim was to reduce the impact of thyme essential oil (EO) on meat sensory properties by using a chemotype with low odor intensity. The pH, color parameters, microbial populations, and sensory properties were assessed during 4 weeks of refrigerated storage. The presence of EO films reduced yeast populations, whereas aerobic mesophilic bacteria, lactic acid bacteria, and enterobacteria were not affected by the presence of the EO in the films. Meat color preservation (a *) was enhanced in the presence of EO, giving a better appearance to the packaged meat. The presence of the chitosan-EO layer reduced water condensation inside the package, whereas packages containing only chitosan had evident water droplets. Thyme odor was perceived as desirable in cooked meat, and the typical product odor intensity decreased by increasing the EO concentration. Further studies should point towards developing oil blends or combinations with natural antimicrobial agents to be incorporated into the film to improve its antimicrobial properties.

## 1. Introduction

Active food packaging may include oxygen scavengers, moisture absorbers, ultraviolet barriers, or compounds that deliver flavoring, antioxidant, or antimicrobial agents [1]. In the context of increasing the demand of multiple hurdle technology to achieve high food safety standards, the development of antimicrobial and antioxidant packaging systems is of great interest. Packaging materials are usually made of synthetic polymers, such as plastic films and multicomponent packages, and they can be used as carriers of active compounds; however the use of edible materials has safety advantages and is more likely to be accepted by consumers. Active edible films can be prepared from plant or animal based proteins, starches, cellulose derivatives, chitin/chitosan, gums, lipids, or mixtures [2]. Chitosan has the ability to form edible and biodegradable films that can carry and release compounds with antimicrobial or antioxidant abilities [3,4,5,6]. 

The use of natural products, such as essential oils (EOs), as food preserving agents is being promoted given the current trend towards green consumerism. Several essential oils (EO) have shown antioxidant properties as well as antimicrobial effects against mold, yeasts, bacteria, and viruses, mainly due to their bioactive components such as flavonoids, terpenes, carotenes, etc. [7,8]. However, EO impairs strong flavor, odor, and even some colors, thus their use is limited to such foods that allow sensory modifications. Given that their direct use in food is limited, other options like EO encapsulation or systems with no contact food-EO may be preferred. Building an active package with no contact between the EO and the food has many advantages like: no taste transfer, reduced organoleptic changes and even distribution of the active compounds in the headspace [1]. There is a clear consumer preference for food with no additives, and so, if the chemical is not added to the food but to the package, it does not need to be declared in the label [8]. Actually, since spoilage occurs mainly on the food surface there is no need to add antimicrobial agents in the bulk of the food, but just to the headspace. EOs may be added to synthetic polymers or sachets as well, however by using edible films there are no safety issues of concern (disintegration and release into the food as well as accidental ingestion of sachets or absorbent pads) [1]. Selecting mild odor EOs may help in reducing the inconvenience of strong flavors.

The antimicrobial activity of EOs is very difficult to compare due to the high variability of the chemical composition of EOs within the same species, affected by many factors (ecological, geographical, and physiological, among others) [9]. EO composition needs to be determined in each study in order to properly define the preservation properties of each EO and conditions of use.

Chitosan edible films containing mild odor thyme EO may be an innovative preservation technique to extend the commercial shelf life of ready to eat meat products, and even replace the use of artificial chemical preservation agents. Although a large number of studies on the antimicrobial effectiveness of EOs are available, very few studies are available on food products. The aim of this study was to evaluate an active packaging system including a layer of chitosan film containing the EO of mild flavored thyme (0%, 0.5%, 1% and 2% EO) for sliced ready to eat (RTE) cooked pork and evaluate the pH, color, evolution of microbial populations, and sensory changes on the RTE meat during refrigerated storage. The main purpose was to evaluate if a layer of chitosan film with thyme EO could be successfully incorporated in a packaging system for RTE meat products to provide an active effect to the packaging and promote an increase in the shelf-life of the cooked meat.

## 2. Materials and Methods 

Essential oil: Thyme (*Thymus vulgaris* L.) commercial essential oil (EO) was purchased from Herbes del Molí (Benimarfull, Alicante, Spain). The company reported that the EO was extracted from the whole plant of organic grown autochthonous thyme by hydro distillation. The company is certified by the Comité de Agricultura Ecológica de la Comunidad Valenciana.

GC-MS and GC-FID Analytical Conditions for oil analysis: The volatile compounds were isolated, identified, and quantified as described in a previous work [10,11], with the only difference being the column used: a Rxi-1301Sil MS (Restek Corporation, 110 Benner Cir, Bellefonte, PA 16823, USA; 30 m, 0.25 mm ID, 1 µm film thickness). Most of the compounds were identified by simultaneously using two different analytical methods [5]: (a) KI, Kováts Index in reference to n-alkanes (C_8_–C_32_); and (b) mass spectra (authentic chemicals and Wiley spectral library collection). Identification was considered to be tentative when it was based on mass spectral data only. Semi-quantification of the compounds was run in a Shimadzu GC-2100 equipped with an FID detector and the same column and conditions as the GC-MS. Quantitative data were obtained electronically from FID area data without using correction factors. All the tests were performed in triplicate.

Film preparation: High molecular weight chitosan of analytical grade was obtained from Sigma Aldrich (800,000 cps; >75% deacetylation degree). The chitosan solution was prepared as follows: 1% chitosan, 1% lactic acid, 0.1% tween, and 0.25% glycerol with different concentrations of EO (0%, 0.5%, 1%, and 2%). To prepare the active packaging, 20 g of each solution were poured into the inner surface of the cover of a Petri dish (90 mm diameter) and were allowed to dry for 72 h at 37 °C, and then the plates were stored at 53% relative humidity until they were used.

Meat material: Commercially cooked ham (400 g pieces, 5 cm diameter, 15 cm length) was used to obtain slices under hygienic conditions. Overall composition, as indicated in the label, was: 18.5% proteins, 1.5% carbohydrates, 1.5% fat, and <0.2% fiber.

**Figure 1:**
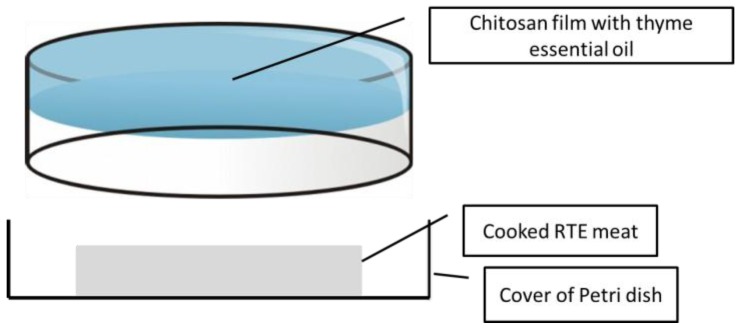
Active packaging design.

**Figure 2:**
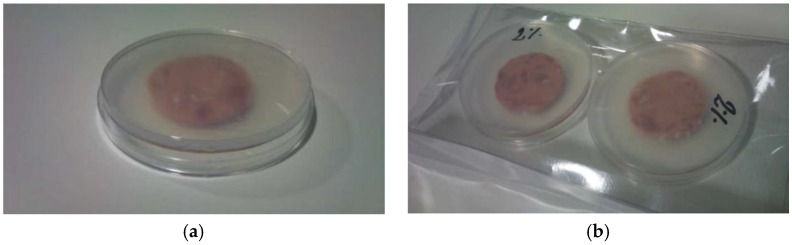
(**a**) Slice of cooked ham inside a Petri dish including a chitosan film, (**b**) final package.

Package design: The basic design was a Petri dish in which the cover had a chitosan film, and one slice of RTE cooked pork was introduced in the opposite cover (no film), and each individual plate was packed in a plastic bag under 50% vacuum (to avoid the collapse of the Petri dish). The package was sealed to avoid uncontrolled release of EO compounds outside of it (Figure 1 and Figure 2). The volatile materials of the EO could be easily released to the headspace of the package without leakage. Packaged samples were kept under refrigeration (3 ± 1 °C) for four weeks, and were sampled weekly for determinations.

Microbial analysis and sampling of meat: Samples were taken weekly for the determination of counts of total aerobic mesophilic bacteria in Plate Count Agar (PCA), incubated at 37 °C for 48 h [5,12]. Mesophilic lactic acid bacteria (MLAB) were determined in MRS agar (Man Rogosa Sharpe) incubated under anaerobiosis at 37 °C for 72 h [5,13]. Enterobacteria were determined in VRBG Agar (Violet Red Bile Glucose), with a double layer to provide microaerophilic conditions, incubated at 37 °C for 24 h. Molds and yeasts were determined in Rose Bengal Agar with Cloramphenicol incubated at 28 °C for 3 days for the yeast count and 5 days for the mold count. Results were expressed as logarithms of colony forming units per gram of cooked meat.

pH determination of meat: The pH was measured in a GLP21 pH-meter (Crison Instruments, Barcelona, Spain) with a punction electrode, and 3 measurements were taken per sample.

Color determination of meat: CIELAB (*Commission internationale de l'éclairage*, L*, a* and b*) color space was used to provide L *, a *, and b * values. A spectrocolorimeter Minolta CM-2022 (Minolta Camera Co. Osaka, Japan) with illuminant D65 and 10° observer was used. A CR-A51 glass (Minolta Camera Co. Osaka, Japan) was put between the sample and the equipment following the American Meat Science Association recommendations (AMSA, 2012). Ten replicates per sample were taken: measurements were taken on both sides of the slices. Hue, Chroma, and ΔE were calculated.

Sensory evaluation of meat: Ten trained judges evaluated the samples for odor, color, and exudates. On each sampling day, 5 closed packages (Figure 2b) from each treatment were provided to the panel for sensory analysis. They were opened by 5 judges and immediately each judge was given one Petri dish containing one slice (Figure 2a). The 10 trained judges evaluated: ham odor, thyme odor, off-odors, color, and presence of exudates. Seven point scales were used for grading the attributes and were described: (a) for all evaluated odors, odor perception was defined as follows 1 = imperceptible, 2 = slight odor perception, 3 = low intensity odor perception, 4 = perceptible odor, 5 = moderate odor intensity, 6 = high odor intensity, 7 = extremely intense odor; (b) for color 1 = extremely light, 2 = moderately light, 3 = slightly light, 4 = regular color, 5 = slightly dark, 6 = moderately dark, 7 = extremely dark; (c) for exudates, 1 = imperceptible presence of exudates, 2 = slight presence of exudates, 3 = low amount of exudates, 4 = evident presence of exudates, 5 = moderate presence of exudates, 6 = high amounts of exudates, 7 = extremely high presence of exudates.

Statistical analysis: The whole experiment was run in duplicate, and triplicate analyses were run for all determinations (10 for color). The IBM SPSS statistics package was used for analysis (SPSS Statistical Software, Inc., Chicago, IL, USA). Microbial counts, color, and pH data were analyzed by an ANOVA with two factors. Thyme EO concentration (4 levels: 0%, 0.5%, 1% and 2%), storage time (5 levels: 0, 7, 14, 21, and 28 days), and their interactions were considered. Tukey’s test was used for mean comparison (*p* < 0.05). Regarding sensory data, a Kruskal-Wallis H test was carried out on the medians.

## 3. Results and Discussion

### 3.1. Essential Oil Composition

Table 1 presents the EO composition. Major compounds of the essential oil were 1,8-cineole, camphor, borneol, α-terpineol, terpinen-4-ol, camphene, *trans*-caryophilene, β-cymene, limonene, and myrcene (present in a range from 3% to 12% of the total profile), accounting for 62.93% of the total profile. There are several chemotypes of thyme, and the present oil was selected based on its mild flavor and because it was a common autochthonous *T. vulgaris.* As can be seen in Table 1, it has no carvacrol or thymol and has a very low content of linalool. Its major component is 1,8-cineole, so it may be considered as a reported endemic Spanish thyme: 1,8-cineole chemotype [14]. The present profile is qualitatively but not quantitatively close to that of rosemary of 1,8-cineole chemotype, also endemic from south eastern Spain [15].

Ballester [16] studied the antimicrobial and antioxidant activities of four endemic thymes from southern Spain, such as *Thymus mastichina* rich in 1,8-cineole (>50% of the total composition of the EO), and compared to the other thymes it showed less antimicrobial effectiveness that the others which were higher in thymol and carvacrol. Ruiz-Navajas et al. [4] studied the antimicrobial effect of two Spanish endemic species of thyme (*Thymus piperella* and *Thymus moroderi*) on several spoilage bacteria. They reported that *T. piperella* had a higher effect than *T. moroderi*, probably due to the higher concentration of carvacrol in *T. piperella*. The thyme used in the present study was not of the same or similar composition to those reported in both studies. The main antimicrobial essential oil compounds are carvacrol, thymol, cinnamaldehyde, methylchavicol, and linalool [8], none of which were present or were present in just small amounts in the studied thyme EO. However, the study is still of interest given that antimicrobial effect cannot be attributed to a single compound but usually to the whole profile [17]. The present composition profile cannot be expected to present such a high antimicrobial activity as that of other chemotypes. As an example, de Oliveira [18] reported a minimum inhibitory concentration for 1,8-cineole against several bacteria to be more than 30 times higher than that of carvacrol for the same bacteria, however the combination of both active compounds presented a synergistic antibacterial effect.

Several food related factors may affect EO antimicrobial activity such as fat content, water activity, and pH, among others. Reduced pH and storage temperature may enhance antimicrobial activity, whereas other factors such as fat content may reduce it [17]. Therefore, effective oil concentrations reported in the literature usually need to be increased when applied in food matrices up to 1%–3% EO, which is the reason why the selected oil concentrations in the film ranged from 0%–2%, whereas 3% was avoided to keep mild odor intensity.

### 3.2. Evolution of Microbial Populations in Packaged RTE Meat during Storage

**Figure 3:**
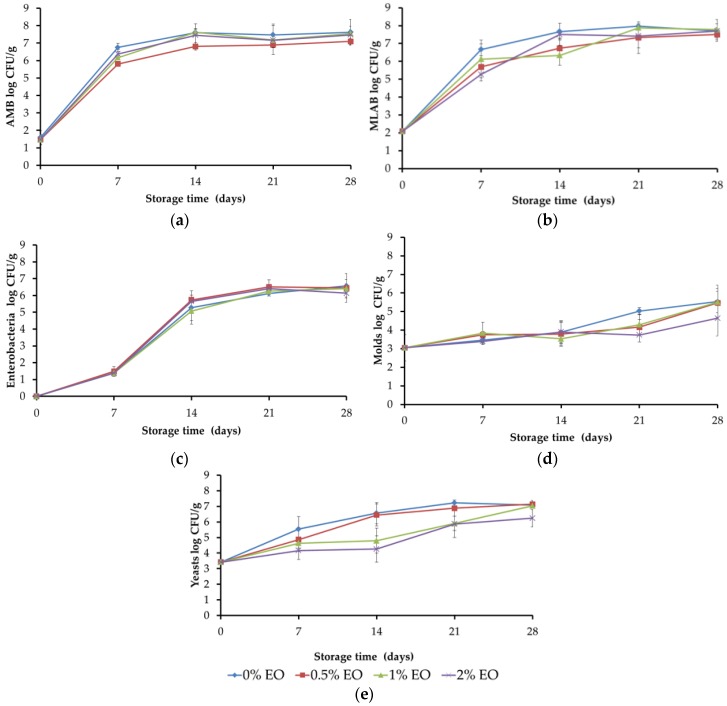
Evolution of (**a**) Aerobic Mesophilic Bacteria (AMB); (**b**) Mesophilic Lactic Acid Bacteria (MLAB); (**c**) Enterobacteria; (**d**) mold and (**e**) yeast counts in RTE meat packaged during refrigerated storage (0% EO: control films; 0.5% EO: films with 0.5% thyme essential oil; 1% EO: films with 1% thyme essential oil; 2% EO: films with 2% thyme essential oil).

Total aerobic mesophilic bacteria (MAB), mesophilic lactic acid bacteria (MLAB), enterobacteria, and molds followed (Figure 3, Table 2) a similar evolution during refrigerated storage despite the presence of thyme EO in the film. Storage time was the only factor significantly affecting the counts of such populations. Yeast counts significantly decreased in the presence of EO in the film in a concentration dependent manner (Figure 3, Table 2). 

Aerobic Mesophilic Bacteria (AMB) counts were under 2 log cfu/g during the first day of the study, reached about 6 log cfu/g after one week of storage, and ended at 7 log cfu/g at the end of the refrigerated storage (Figure 3). Although no significant differences were detected, microbial counts in the control sample (0% EO) were always higher than those of samples packaged in the presence of EO.

Several authors studied chitosan films with thyme EO obtaining similar results to ours [19], however others [6,7] reported higher antimicrobial effectiveness of chitosan incorporated with thyme EO. In studies reporting higher effectiveness, meat samples were immersed in chitosan solutions, so direct contact of the EO with the meat was achieved. In the present study, the lack of effectiveness against most of the studied microbial populations may be due to the combined effect of no contact between the chitosan containing EO and the meat, together with the composition of the oil (1,8-cineole chemotype). Much higher antimicrobial effectiveness was observed in studies using thymes higher in carvacrol and thymol either in vitro or in direct contact with the cooked meat [5,6,16].

Scarce or little effect of thyme EO presence has been observed on mesophilic LAB counts, although counts tended to be higher in the control packages. After only seven days of storage, MLAB populations were significantly lower in RTE packaged with EO at all concentrations. As storage time increased, MLAB populations became similar for all packaging conditions. Other studies have reported that *Lactobacillus plantarum* was scarcely affected by chitosan films containing thyme and oreganum EOs [19]. In a study on poultry products, LAB decreased in 4 log/cfu of LAB when chitosan films (1.5% chitosan and 0.2% thyme EO) were formed on the meat surface after immersion in the chitosan solution [20]. In contrast, Petrou et al. [21] reported that oreganum EO addition to chitosan films did not enhance antimicrobial activity, as observed in the present study.

Enterobacteria were not detected during the first day of the study, were detected after one week of storage around 1 log cfu/g, and at about 5 log cfu/g at the end of the study (Figure 3). Regarding enterobacteria, no antimicrobial effect of thyme EO was observed, which is consistent with the high resistance of gram negative bacteria against EOs. However, other authors reported a decrease of 2.5 to 4 log cfu/g of enterobacteria when chitosan was used either alone or containing oreganum EO [20,21]. Chitosan diluted in acetic acid had an antibacterial effect against enterobacteria in meat when meat samples were immersed in the chitosan solution [21].

Mold initial counts were 3 log cfu/g in all batches and were not significantly affected by EO presence (Table 2). Mold counts increased with storage time. Yeast populations were affected by the presence of thyme EO in the chitosan film (Table 2), and the yeast counts decreased as a function of the EO dose in the film, especially during the first 21 days of storage. Others reported reduced mold (not affected in the present study) and yeast in treatments combining chitosan and thyme EO [22]; and a 2 log cfu/g yeast decrease when chitosan was incorporated with oreganum EO [21]. The present result is highly relevant as yeasts are commonly involved in the spoilage of cooked meat products [23].

Given that yeasts were inhibited in the presence of EO, it may be assumed that EO was in fact released from the film, so the present results point to a scarce antibacterial activity but a promising activity against yeast from this 1,8-chemotype thyme. The reduction of mold and yeast counts in active packages containing EOs has been previously reported in mangos in packages containing sachets with oregano and lemongrass [24], and in mozzarella cheese in packages containing rosemary and thyme oils [25].

### 3.3. Evolution of the pH of RTE Meat during Storage in Active Packaging

The pH of RTE meat packaged in the presence of EO (Table 2 and Table 3) was lower than that of the control. Other authors reported increased pH in control packaged poultry meat [26] compared to that including EO, whereas in the present study there is a pH reduction in RTE meat packaged in the presence of EO. All packaged meats experienced a pH decrease until the 21st day of storage, probably due to increased lactic acid bacteria populations (Figure 3). However, as mesophilic microbial populations, molds, and yeasts significantly kept increasing until the 28th day of storage, an incipient proteolysis linked to their metabolic activity may have accounted for the increased pH at the 28th day of storage.

### 3.4. Evolution of Color Parameters of Packaged RTE Meat during Storage

As can be seen in Figure 2, cooked ham slices were not completely uniform in color, as they were modified by the presence and orientation of muscular tissues; this fact is reflected in the variability of the results (Table 4). 

Regarding color parameters, no differences were reported for L * (Table 4) due to EO addition, although RTE meat packaged in the presence of EO did not have significant higher values of L *, as reported by Petrou et al. [21] in poultry meat packaged with EO. L * was significantly reduced with storage time. Cooked ham b * value showed a tendency to increase with EO addition to the film, however only ham packaged in the system with 2% EO showed significantly higher b * than the control. Storage time significantly decreased b * values by less than one unit. Coordinate a * was better preserved (Table 4) and even enhanced (approximately one unit for the 2% EO batch) by the EO addition to the films in a concentration dependent manner (Table 5 and Table 6). Hue, Chroma, and ΔE parameters were calculated (Table 4). The larger the hue values, the lower the red color, and this parameter is useful to indicate shifts in color over time. In Table 2 and Table 4 it can be clearly seen that storage time as well as the presence of EO enhanced redness preservation (as both significantly decreased Hue values). Regarding Chroma value, representing the intensity of the principle Hue, these values increased with the presence of EO in the package (Table 2 and Table 4). Storage time unevenly affected the Chroma parameter, which ranged within a narrow range of values (shifts under 5%). ΔE allows the determination of the total color change over storage time, and the average color of the cooked ham at day zero was taken as reference value for the calculations. Color differences significantly increased with storage time, and were unevenly affected by the presence of EO in the package. 

Redness preservation due to the use of edible films with thyme EO has been previously reported in ground beef patties [27], and it has been linked to the control of oxidative changes due to the presence of EO. In the present study, it may also be related to the reduced presence of exudates, and also reduced free water in the package perceived by consumers. It is a general fact that during meat refrigerated storage L * tends to increase and a * values tend to decrease due to the oxidation of hemopigments [28]. Kanatt et al. [29] reported antioxidant properties of chitosan films on meat, and in the present study even the control group, which contained chitosan without EO, presented decreased L * values (approximately 5 units) and maintained a * values, thereby proving the antioxidant effect of chitosan. The antioxidant properties of chitosan were improved by EO addition as color parameters were better preserved in the presence of EOs.

The sensory panel detected significant color changes with storage time and no significant differences between slices packaged with different EO content (Table 5 and Table 6), however, they reported decreased color intensity with storage time. This observation, which is opposed to objective color measurements, may be related to the misleading effect of the presence and orientation of muscular tissue, as well as the fact that there was no standard to compare with. This is a clear example of the need for objective measurements in order to detect color changes over time.

### 3.5. Sensory Charactaristics of Packaged RTE Meat

Sensory evaluation was necessary as not all foods are suitable to be combined with EO odor and flavor. In Table 5 and Table 6, results of the sensory analysis are presented. Storage time was the main factor affecting judges’ perception of all descriptors, with exudates, color, and ham odor being the most affected parameters (*p* > 0.01), whereas off-flavors and thyme flavor were scarcely affected (*p* < 0.05). The presence of thyme EO affected ham odor, thyme odor, and the presence of exudates, whereas no significant differences were detected for off-flavors and perceived color intensity. Ham odor decreased as EO presence in the package increased, whereas thyme odor increased with increased presence of EO. Panelists indicated that the perceived thyme odor was desirable, and was not considered an off-flavor for this type of product, which is commonly available with fine herbs and similar odor intensity. The evolution of the odor was expected as related to meat ageing and the presence of an EO that poses high aromatic intensity, however the most relevant result is the reduced presence of exudates in those packaging systems containing EO (Table 5). This may be due to the modifications in the chitosan film structure caused by the presence of EO which may have enhanced moisture absorption. Ojagh et al. [10] reported that the presence of cinnamon EO in chitosan films reduced film moisture and enhanced film permeability to water vapor. The reduction of exudates linked to EO incorporation in the films may be related to enhanced water solubility of the films when EO are added as previously reported in protein based films [30]. The liquid exuded from meat may have a negative impact on the sensory aspect as well as potentially enhance proliferation of pathogens or spoilage microorganisms, acting as a microbial media [1], so the reduction of exudates enhances the product image and potentially food safety.

Regarding sensory scores reported in other studies from the scientific literature of products packaged in the presence of EO, results were uneven. Whereas cheeses were preferred with no EO in the package [25], chicken drum-sticks were preferred with pads containing 1.5% oregano EO in the sachets as they provided a characteristic desirable odor [26], and good sensory scores were obtained for bologna with chitosan films including oregano EO [31]. In the last study, scores decreased with increasing the concentration of EO, and direct application on bologna of up to 45 ppm of EO would be acceptable for consumers.

## 4. Conclusions 

We designed a model package with the inclusion of a layer of chitosan incorporated with thyme EO with the advantage of possible automation. Sachets and pads may be also incorporated but they need to be manually added to the packages, whereas applying a coating to a plastic layer or side of the package may provide technical benefits. Edible films with EOs are safe for consumers and there is no need to use warnings labels to not eat the package.

Regarding the use of the active packaging with thyme EO (1,8-cineole chemotype) in RTE cooked pork, higher antimicrobial activity of the EO was demonstrated against yeast, whereas the other studied microbial populations were not affected by the presence of the EO. The presence of the chitosan-EO layer avoided water condensation, whereas the packages containing only chitosan had evident water droplets. The most remarkable sensory effects were observed for odor, with ham odor intensity decreasing with increased EO concentration, as a result of a masking process produced by thyme odor. However, thyme odor was perceived as desirable for this RTE. Other issues need to be addressed in future works such as EO migration kinetics, duration of the activity of the films, and, when brought to the industrial scale, the packaging processing effect on film retention and properties. In the present work, our aim was to reduce the impact of EO on meat sensory properties by using thyme from a chemotype with mild odor, which happened to have low content of antimicrobial compounds (thymol and carvacrol). Even under such conditions, yeast populations were controlled, color preservation was enhanced, and exudates in the package were reduced, which gave a better appearance to the packaged meat. In future studies, combining EOs with organic acids or salts, or even with isolated active compounds, should also be explored.

## Figures and Tables

**Table 1:** Principal constituents of *Thymus vulgaris* essential oil, their relative percentages of the total chromatogram area, and Kovats Index.

Composition	Id. ^1^	KI	*Thymus vulgaris* (%)
α-Pinene	KI, W	937	1.12
β-Pinene	KI, W	948	2.85
**Camphene**	**KI, W**	**970**	**4.78**
Sabinene	KI, W	993	1.94
β-Pinene	KI, W	1000	2.76
**Myrcene**	**KI, W**	**1003**	**3.27**
1-Octen-3-ol	KI, W	1023	0.78
α-Terpinene	KI, W	1035	0.87
**Limonene**	**KI, W**	**1046**	**3.43**
**β-Cymene**	**KI, W**	**1051**	**3.74**
**1,8-Cineole**	**KI, W**	**1062**	**12.30**
γ-Terpinene	KI, W	1074	2.26
Terpinolene	KI, W	1103	0.45
*trans*-Sabinene hydrate	KI, W	1118	1.30
Linalool	KI, W	1142	1.90
*cis*-Sabinene hydrate	KI, W	1154	0.87
Terpineol	KI, W	1174	0.31
*trans*-Pinocarveol	KI, W	1199	1.36
Verbenol	KI, W	1207	1.32
**Camphor**	**KI, W**	**1215**	**11.23**
**Terpinen-4-ol**	**KI, W**	**1228**	**5.50**
**Borneol**	**KI, W**	**1238**	**8.87**
**α-Terpineol**	**KI, W**	**1250**	**5.83**
Myrtenol	KI, W	1260	0.23
*cis*-Carveol	KI, W	1263	0.25
Citronellal	KI, W	1279	0.17
Linalyl acetate	KI, W	1284	0.38
Verbenone	KI, W	1294	0.23
Bornyl acetate	KI, W	1331	2.38
α-Terpenyl acetate	KI, W	1388	0.93
β-Bourbonene	KI, W	1416	0.63
Bornyl acetate	KI, W	1421	1.91
α-Gurjunene	KI, W	1436	0.13
**trans-Caryophyllene**	**KI, W**	**1460**	**3.98**
Alloaromadendrene	KI, W	1497	2.03
Germacrene-D	KI, W	1522	0.57
α-Murolene	KI, W	1525	0.73
Bicyclogermacrene	KI, W	1537	1.65
δ-Cadinene	KI, W	1545	2.74
Hedycariol	KI, W	1616	1.12

^1^ “KI, W” means that identification was based on Kováts indexes (KI) and comparison with the Wiley library (W).

**Table 2:** Effect of (A) thyme EO concentration (4 levels: 0%, 0.5%, 1% and 2%) and (B) storage time (5 levels: 0, 7, 14 and 28 days) on microbial counts. pH and color parameters in RTE meat packaged during refrigerated storage.

Variables	A = Thyme EO Concentration ^1^					B = Storage Time (Days) ^1^						A × B
	0%	0.5%	1%	2%	*F-*Value	0	7	14	21	28	*F*-Value	*F*-Value
AMB	a	a	a	a	1.568NS	a	b	c	c	d	1882.396 **	2.619 *
MLAB	a	a	a	a	1.269NS	a	b	c	d	d	609.583 **	3.159 *
Enterobacteria	a	a	a	a	1.137NS	a	d	c	d	d	839.761 **	0.963NS
Molds	a	a	a	a	1.480NS	a	ab	b	b	c	17.121 **	0.310NS
Yeasts	b	b	a	a	24.332 **	a	b	b	c	c	101.708 **	3.808 **
pH	d	c	a	b	77.826 **	c	c	b	a	b	151.656 **	10.402 **
L *	a	a	a	a	1.733NS	c	b	b	b	a	65.752 **	9.410 **
a *	a	a	b	b	16.269 **	ab	a	b	c	c	25.657 **	3.426 **
b *	a	ab	ab	b	1.931 *	bc	bc	c	b	a	30.362 **	5.169 **
C	a	ab	bc	c	10.065 **	ab	ab	bc	c	a	5.190 **	1.516NS
H	bc	c	a	ba	8.391 *	c	d	cd	b	a	53.273 **	7.262 **
Delta E	b	ab	a	ab	11.260 **	a	b	b	b	c	67.798 **	10.303 **

^1^ Different letters, in the same line, indicate significant differences *p* < 0.05; AMB: Aerobic Mesophilic Bacteria; MLAB: Mesophilic Lactic Acid Bacteria; NS, not significant; * Significant at *p* < 0.05; ** Significant at *p* < 0.01.

**Table 3:** pH values (mean ± standard deviation) in RTE meat packaged during refrigerated storage (0% EO: control films; 0.5% EO: films with 0.5% thyme essential oil; 1% EO: films with 1% thyme essential oil; 2% EO: films with 2% thyme essential oil).

pH	Days of Storage				
	0	7	14	21	28
0% EO	6.35 ± 0.01	6.31 ± 0.01	6.23 ± 0.04	6.19 ± 0.04	6.33 ± 0.08
0.5% EO	6.35 ± 0.01	6.30 ± 0.02	6.14 ± 0.07	5.97 ± 0.04	6.26 ± 0.06
1% EO	6.27 ± 0.01	6.26 ± 0.07	6.08 ± 0.04	5.82 ± 0.02	6.00 ± 0.01
2% EO	6.27 ± 0.01	6.27 ± 0.01	6.15 ± 0.01	5.90 ± 0.08	6.04 ± 0.04

**Table 4:** Color parameters (mean ± standard deviation) in RTE meat packaged during refrigerated storage (0% EO: control films; 0.5% EO: films with 0.5% thyme essential oil; 1% EO: films with 1% thyme essential oil; 2% EO: films with 2% thyme essential oil). * Delta E, total color differences between 0% EO (day 0).

**Lightness**	**Days of Storage**				
	**0**	**7**	**14**	**21**	**28**
0% EO	66.28 ± 1.71	62.18 ± 0.92	59.82 ± 1.90	60.98 ± 1.85	61.85 ± 1.65
0.5% EO	64.94 ± 1.25	63.14 ± 1.91	63.53 ± 1.47	62.64 ± 0.90	59.47 ± 1.48
1% EO	66.79 ± 1.82	64.70 ± 1.10	62.81 ± 1.26	63.97 ± 1.11	60.26 ± 1.65
2% EO	66.10 ± 1.07	59.56 ± 1.36	61.88 ± 0.61	61.15 ± 0.45	61.66 ± 2.82
a * (Redness/Greenness)	**Days of Storage**				
	**0**	**7**	**14**	**21**	**28**
0% EO	6.03 ± 0.40	5.79 ± 0.24	6.13 ± 0.35	6.73 ± 0.58	6.27 ± 0.75
0.5% EO	6.28 ± 0.65	5.49 ± 0.53	5.41 ± 0.68	7.08 ± 0.47	6.91 ± 0.38
1% EO	6.52 ± 0.23	6.03 ± 0.23	7.08 ± 0.80	6.89 ± 0.38	7.60 ± 0.48
2% EO	6.45 ± 0.32	6.39 ± 0.75	6.54 ± 0.46	7.44 ± 0.74	7.52 ± 0.51
b * (Yellowness/Blueness)	**Days of Storage**				
	**0**	**7**	**14**	**21**	**28**
0% EO	8.31 ± 0.30	8.27 ± 0.27	8.82 ± 0.78	7.67 ± 0.67	7.24 ± 0.25
0.5% EO	8.27 ± 0.23	8.46 ± 0.45	9.09 ± 0.94	7.97 ± 0.41	6.91 ± 0.25
1% EO	8.15 ± 0.42	8.55 ± 0.73	7.84 ± 0.21	8.68 ± 0.36	7.51 ± 0.38
2% EO	8.57 ± 0.24	8.61 ± 0.03	8.64 ± 0.59	8.23 ± 0.27	7.70 ± 0.42
**C (Chroma)**	**Days of Storage**				
	**0**	**7**	**14**	**21**	**28**
0% EO	10.28 ± 0.23	10.10 ± 0.23	10.48 ± 0.58	10.22 ± 0.88	9.93 ± 0.33
0.5% EO	10.40 ± 0.33	10.10 ± 0.48	10.58 ± 0.95	10.63 ± 0.32	9.78 ± 0.22
1% EO	10.41 ± 0.43	10.47 ± 0.63	10.52 ± 0.56	11.08 ± 0.48	10.69 ± 0.54
2% EO	10.73 ± 0.31	10.89 ± 0.45	10.79 ± 0.64	11.09 ± 0.52	10.76 ± 0.64
**h (Hue)**	**Days of Storage**				
	**0**	**7**	**14**	**21**	**28**
0% EO	54.02 ± 2.47	55.00 ± 1.58	55.09 ± 3.50	48.65 ± 0.67	49.53 ± 5.14
0.5% EO	52.82 ± 2.77	57.04 ± 2.88	59.22 ± 3.69	48.67 ± 2.88	45.04 ± 2.31
1% EO	51.46 ± 1.09	54.72 ± 2.37	47.78 ± 3.33	51.57 ± 0.99	44.67 ± 1.50
2% EO	53.04 ± 1.37	53.03 ± 3.24	53.08 ± 2.08	47.91 ± 2.07	45.69 ± 0.90
Delta E *	**Days of Storage**				
		**7**	**14**	**21**	**28**
0% EO		4.13 ± 0.92	6.53 ± 1.27	5.45 ± 1.14	4.65 ± 1.41
0.5% EO		3.28 ± 0.80	3.19 ± 1.14	3.88 ± 1.01	7.02 ± 1.47
1% EO		1.85 ± 0.20	3.82 ± 1.11	2.55 ± 0.56	6.30 ± 0.61
2% EO		6.79 ± 0.70	4.48 ± 0.79	5.37 ± 1.71	4.94 ± 0.68

**Table 5:** Sensory descriptors (median) of RTE meat packaged during refrigerated storage (0% EO: control films; 0.5% EO: films with 0.5% thyme essential oil; 1% EO: films with 1% thyme essential oil; 2% EO: films with 2% thyme essential oil). Descriptors assessed on a 7 point scale.

**Ham Odor**	**Days of Storage**				
	**0**	**7**	**14**	**21**	**28**
0% EO	5.0	5.5	5.0	3.0	3.0
0.5% EO	4.0	4.0	3.0	2.0	5.0
1% EO	2.0	3.5	3.0	3.0	4.0
2% EO	1.0	1.0	2.0	1.5	2.0
**Off-Flavors**	**Days of Storage**				
	**0**	**7**	**14**	**21**	**28**
0% EO	1.0	1.0	1.0	2.0	1.0
0.5% EO	1.0	1.0	1.0	1.0	1.0
1% EO	1.0	1.0	2.0	1.0	1.0
2% EO	1.0	1.0	1.0	1.0	1.0
**Thyme Odor**	**Days of Storage**				
	**0**	**7**	**14**	**21**	**28**
0% EO	1.0	1.0	1.0	2.0	1.0
0.5% EO	3.0	2.0	2.0	3.0	1.0
1% EO	5.0	2.5	3.0	4.0	3.0
2% EO	6.0	6.0	5.5	5.5	4.0
**Color**	**Days of Storage**				
	**0**	**7**	**14**	**21**	**28**
0% EO	4.0	3.5	3.0	3.0	3.0
0.5% EO	4.0	4.0	3.5	3.0	2.0
1% EO	4.0	3.0	4.0	3.0	2.0
2%EO	4.0	4.0	3.0	3.5	2.0
**Exudates**	**Days of Storage**				
	**0**	**7**	**14**	**21**	**28**
0%EO	1.0	2.5	6.5	4.0	4.0
0.5% EO	1.0	1.0	1.0	1.0	2.0
1% EO	1.0	1.0	1.0	1.0	2.0
2%EO	1.0	1.0	1.0	1.0	2.0

**Table 6:** Significance of the effect of factorsthyme EO concentration and storage time on sensory attributes in RTE meat packaged during refrigerated storage.

Variables	Thyme EO Concentration	Storage Time
	*Sig ^1^*	*Sig ^1^*
Ham odor	**	**
Off-flavors	NS	*
Thyme odor	**	*
Color	NS	**
Exudates	*	**

^1^ According to Kruskal-Wallis H; * Significant at *p* < 0.05; ** Significant at *p* < 0.01; NS, not significant.
